# Emergence of *Escherichia coli* Hybrid Pathotype ETEC/STEC: A Macroepidemiological Approach of Molecular Characterization in the United States Swine Population

**DOI:** 10.1155/tbed/8370419

**Published:** 2026-07-01

**Authors:** Elisa De Conti, Guilherme Cezar, Thuc Quyen Le, Meghana Korada, Kinath Rupasinghe, Hemant K. Naikare, Angela Pillatzki, Craig L. Bowen, Melanie Prarat, Franco Matias Ferreyra, Rodger Main, Paul Yeske, Mark Schwartz, Beth Thompson, Peter Schneider, Samuel Copeland, Luc Dufresne, Daniel Boykin, Megan Niederwerder, Deborah Murray, Brigitte Mason, Corrine Frugé, William Hollis, Rebecca Robbins, Thomas Petznick, Kurt Kuecker, Lauren Glowzenski, Brooke Kitting, Gustavo Silva, Bret Crim, Eric Burrough, Phillip Gauger, Michael Zeller, Sara McReynolds, Darin Madson, Mary Thurn, Paulo Fioravante, Albert Rovira, Rob McGaughey, Jamie Retallick, Jon Greseth, Darren Kersey, Travis Clement, Jane Christopher-Hennings, Dennis Summers, Kenitra Hendrix, James Lyons, Joseph Boyle, Leticia Linhares, Edson Magalhães, Christopher Rademacher, Daniel Linhares, Marcelo Almeida, Giovani Trevisan

**Affiliations:** ^1^ Veterinary Diagnostic and Production Animal Medicine, Iowa State University, Ames, Iowa, USA, iastate.edu; ^2^ Veterinary Population Medicine, University of Minnesota, Saint Paul, Minnesota, USA, umn.edu; ^3^ Veterinary and Biomedical Sciences Department, South Dakota State University, Brookings, South Dakota, USA, sdstate.edu; ^4^ College of Veterinary Medicine, Purdue University, West Lafayette, Indiana, USA, purdue.edu; ^5^ Ohio Animal Disease and Diagnostic Laboratory, Reynoldsburg, Ohio, USA; ^6^ Kansas Veterinary Diagnostic Laboratory, Kansas State University, Manhattan, Kansas, USA, k-state.edu; ^7^ Swine Vet Center, PA, St. Peter, Minnesota, USA; ^8^ Schwartz Farms, Sleepy Eye, Sleepy Eye, Minnesota, USA; ^9^ South Dakota Animal Industry Board, Pierre, South Dakota, USA; ^10^ Innovative Agricultural Solutions, LLC, Ames, Iowa, USA; ^11^ Prestage Farms, St. Pauls, North Carolina, USA; ^12^ Swine Veterinary Partners, Quebec, California, USA; ^13^ Smithfield Foods, Smithfield, Virginia, USA; ^14^ Swine Health Information Center, Ames, Iowa, USA; ^15^ New Fashion Pork, Jackson, Minnesota, USA; ^16^ Country View Family Farms, Hatfield, Pennsylvania, USA; ^17^ The Maschhoffs, LLC, Carlyle, Illinois, USA; ^18^ Carthage Veterinary Service Ltd., Carthage, Illinois, USA; ^19^ PIC, Hendersonville, Tennessee, USA; ^20^ Independent Veterinarian, Omaha, Nebraska, USA; ^21^ The Hanor Company, Enid, Oklahoma, USA; ^22^ Pipestone Veterinary Services, Pipestone, Minnesota, USA; ^23^ Seaboard Foods, Guymon, Oklahoma, USA; ^24^ Kansas Department of Agriculture, Manhattan, Kansas, USA; ^25^ Department of Animal Science, Iowa State University, Ames, Iowa, USA, iastate.edu

## Abstract

*Escherichia coli*–associated diseases continue to be a significant concern in swine health. Disease may occur when *E. coli* strains harbor specific virulence factors. The combination of virulence factors detected in a single isolate forms a virotype, which can be grouped into pathotypes based on mechanisms of pathogenicity. This study compiled *E. coli* polymerase chain reaction (PCR) sample‐level data from six major U.S. veterinary diagnostic laboratories (VDLs) to evaluate trends in *E. coli* virulence factors, virotypes, and pathotypes from porcine cases. The dataset contained diagnostic results for *E. coli* virulence factors detected by PCR, including attachment genes (fimbriae and adhesins) and toxin genes (heat‐labile, heat‐stable, and Shiga‐like toxins). Between 2008 and 2025, major shifts were observed in the detection of key *E. coli* virulence factors. Temporal trend analysis using the Mann–Kendall test showed increasing detection of the virulence factors F18, F41, STa, Stx2, and Stx2e, while F4, AIDA, Paa, and EAST1 decreased over time. Similar patterns were observed among isolates classified as potentially pathogenic, with increases in F18, F41, STa, Stx2, and Stx2e and decreases in F4, AIDA, Paa, and EAST1 detection. Among pathotypes, Hybrid enterotoxigenic *E. coli* (ETEC)/STEC had an increase in detection over time, whereas ETEC, STEC, and isolates classified as not potentially pathogenic decreased. This large‐scale diagnostic dataset reveals a notable shift in the *E. coli* pathogenic profile in swine over the past decade, characterized by an increasing detection of the hybrid ETEC/STEC pathotype and evolving virotype compositions.

## 1. Introduction

*Escherichia coli* is a Gram‐negative bacterium that inhabits the pig’s microbiota [1]. Certain strains of *E. coli* can cause a wide range of diseases in pigs [2]. The most common diseases include neonatal diarrhea, post‐weaning diarrhea, and edema disease, which are associated with distinct *E. coli* virotypes [3]. Neonatal diarrhea primarily affects piglets in their first week of life and is often caused by enterotoxigenic *E. coli* (ETEC) strains that express fimbriae, such as F4 (K88) or F5 (K99), along with enterotoxins, such as Sta. In contrast, post‐weaning diarrhea and edema disease typically occur in pigs one to 3 weeks after weaning and are associated with ETEC and Shiga toxin‐producing *E. coli* (STEC), respectively [3, 4]. The ETEC strains in post‐weaning diarrhea commonly express virulence factors such as F18ac and STb, while STEC strains implicated in edema disease harbor F18ab and Stx2e [2].

While *E. coli* can be associated with various diseases; it may also be present in healthy pigs [5]. Therefore, the characterization of *E. coli* virulence factors has become essential for the accurate diagnosis of potentially pathogenic strains [6]. When a clinical sample arrives at the laboratory with a suspicion of an *E. coli* infection, the most common approach is to initially process the sample through a general bacteriological culture for the isolation of enteric pathogens. Once *E. coli* is isolated, further diagnostic testing is important to determine whether the isolate harbors specific virulence factors that characterize a strain as pathogenic or not [7]. Polymerase chain reaction (PCR) has been developed as a valuable tool that utilizes specific primers and probes capable of targeting one or multiple genes, including those previously identified as virulence factors [8, 9]. Moreover, next‐generation sequencing, particularly whole‐genome sequencing, has emerged as a tool for the characterization of *E. coli* isolates, enabling simultaneous detection of virulence genes, antimicrobial resistance determinants, and phylogenetic profiling [10–12].

PCR genotyping is one diagnostic method used to detect *E. coli*‐specific genes encoding virulence factors (e.g., F18 and Stx2e). Based on the combination of these detected genes, samples are characterized into virotypes (e.g., F18:Stx2e), which represent specific virulence profiles. These virotypes, in turn, can then be grouped into pathotypes (e.g., STEC) based on the mechanisms by which the *E. coli* may cause disease in pigs [3]. This approach is important for diagnostic purposes as it enables the identification of pathogenic *E. coli* based on their virulence gene profiles, distinguishing them from nonpathogenic, commensal strains.

In the last couple of years, *E. coli* disease cases, particularly in the post‐weaning period, have become a reemerging concern in the United States [13]. A recent study reported that the wean‐to‐finish mortality for groups without clinically apparent enteric disease was 9.4%, while mortality in groups with clinical disease typical of *E. coli* was 11.1% [14]. Other recent publications demonstrated a high increase in the number of confirmed cases of post‐weaning colibacillosis, escalating from around 6% of the porcine enteric cases in 2010 to over 12% in 2021 [15, 16]. The hybrid pathotype STEC/ETEC (strains carrying F18 fimbria genes, heat‐labile and/or heat‐stable toxin genes of ETEC, and the *Stx2e* gene of STEC) is an emerging concern [15, 17–19]. Experimental infection confirmed the ability of this pathotype to cause severe watery diarrhea without edema disease in post‐weaning pigs [20]. The increasing detection of virulent *E. coli* strains associated with disease in pig herds underscore the need for ongoing monitoring at the national level. Moreover, from an epidemiological perspective, the characterization of *E. coli* virotypes and pathotypes enable a more robust trend analysis, providing science‐driven information that can be used to support the decision‐making process in controlling this bacterium.

In this scenario, an organized hub for data collection, collation, analysis, and generation of epidemiological information can aid in a better understanding of the distribution patterns of *E. coli* virulence factors, virotypes, and pathotypes. The increasing challenge posed by *E. coli* in swine production emphasizes the importance of developing a comprehensive epidemiological tool, similar to what has been provided for other swine pathogens [21–23]. Therefore, the objective of this study was to describe the macroepidemiological trends in the detection of *E. coli* virulence factors, virotypes, and pathotypes while establishing a centralized data hub for real‐time reporting and ongoing characterization of *E. coli* molecular trends over time.

## 2. Materials and Methods

### 2.1. Data Source

*E. coli* gel‐based PCR genotyping results and associated metadata (i.e., received date, farm type, pig age, specimen, and site state) were retrieved from Iowa State University (ISU), University of Minnesota (UMN), and Kansas State (KS) respective veterinary diagnostic laboratories (VDLs), and from South Dakota State University Animal Disease Research and Diagnostic Laboratory (SDSU ADRDL), Ohio Animal Disease Diagnostic Laboratory (ADDL), and Purdue University ADDL. Participant laboratories integrated the network, contributing data for the Swine Disease Reporting System (SDRS; https://www.fieldepi.org/SDRS). Briefly, SDRS aggregates data from multiple veterinary laboratories, performs data analysis, and reports findings in near real time [23]. Currently, the SDRS integrates porcine reproductive and respiratory syndrome virus (PRRSV), porcine epidemic diarrhea virus (PEDV), porcine deltacoronavirus (PDCoV), transmissible gastroenteritis virus (TGEV), *Mycoplasma hyopneumoniae*, porcine circovirus type 2 (PCV2), porcine circovirus type 3 (PCV3), and influenza A virus (IAV) detection by PCR or reverse transcription PCR (RT‐PCR) testing assays, plus PRRSV open reading frame‐5 (ORF‐5) gene Sanger sequencing [21–24].

### 2.2. Data Management

#### 2.2.1. Data Handling

Following a methodology similar to that described in previous studies, anonymized *E. coli* gel‐based PCR genotyping diagnostic testing and testing results data were retrieved from the six VDLs [21, 22, 25]. The dataset included information on submission data (i.e., received date, farm type, specimen, species, and state from where the samples were submitted), tests, and test results. All data were organized and collated into a standardized format. A modification from previous studies was applied to organize the data at the sample level, i.e., unique sample ID. Each unique sample ID had one set of results for each *E. coli*‐targeted gene tested by PCR.

### 2.3. Data Collection

**Figure 1:**
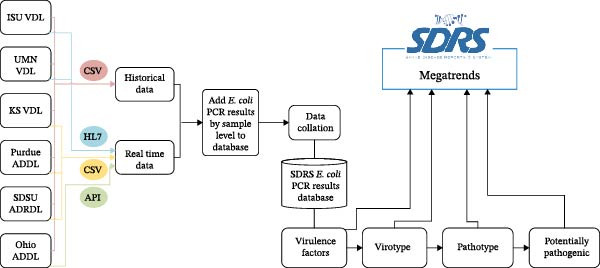
The *Escherichia coli* PCR genotyping data collection, collation, and organization. ADDL, animal disease diagnostic laboratory; API, application programing interface; CSV, comma‐separated values; HL7, health level seven; ISU, Iowa State University; KS, Kansas State; SDSU ADRDL, South Dakota State University animal disease research and diagnostic laboratory; SDRS, swine disease reporting system; UMN, University of Minnesota; VDL, veterinary diagnostic laboratories.

The clientele‐anonymized PCR submission data results recovered from the six VDLs were collated into a standardized format at the submission and sample (sample ID) level using a Python script [26]. A specialized Microsoft SQL Server database hosted on the secure and scalable servers of the ISU Veterinary Diagnostic and Production Animal Medicine Department (ISU VDPAM) was developed to efficiently receive, organize, and collate the *E. coli* PCR genotyping data. The historical data were provided by participating VDLs in comma‐separated values (CSV) format, through a one‐time data pooling conducted between June 2024 and May 2025, spanning a data time frame from 2000 to 2024. After the historical data pooling, the laboratories kept sharing prospective data in different formats. For prospective sharing, ISU VDL and UMN VDL utilized Health Level Seven (HL7) messaging for real‐time data transmission. SDSU ADRDL and Purdue ADDL submitted CSV files every month, while KS VDL shared weekly CSV files. The Ohio ADDL provided an application programing interface (API) connection that was configured for daily “GET” calls for prospective data collection (Figure 1).

#### 2.3.1. Data Organization and Visualization

Cases identified as originating from porcine species were retained and used to analyze trends in *E. coli* virulence factors, virotype, and pathotype over time. The results were collated into three levels: *E. coli* PCR genotyping (i.e., virulence factors tested, i.e., result for each PCR target tested), virotype (i.e., positive virulence factors detected within an isolate), and pathotype (i.e., classification of the potential mechanism of disease causation of the virotype). Moreover, the pathotypes were further classified as potentially pathogenic or not. For analyzing trends in detection, only data from January 2008 to December 2025 were retained. Analyses started in 2008 due to better data completeness captured and shared by the participant VDLs. Cases explicitly identified as research were excluded. Additionally, cases not labeled as research but containing more than 10 submitted samples within one case were also treated as research cases and excluded from the analysis. The results were displayed in charts generated with ggplot2 in R [27] for data visualization.

#### 2.3.2. Virulence Factors

All *E. coli* PCR targets (i.e., virulence factors) tested were reported as positive or negative. When a target was not tested, it was reported as not being tested. The PCR targets had two categories: the ones from attachment genes, (i.e., fimbriae [F18, F41, F4 (K88), F5 (K99), and F6 (987P)] or adhesins [AIDA, Eae (Intimin), and Paa]) and the ones from toxin genes (EAST1, LT, STa, STb, Stx1, Stx2, and Stx2e).

#### 2.3.3. Virotype

All positive virulence factors detected in a single sample were compiled into the virotype. The order of the collation was first attachment genes, arranged in an alphabetical order, followed by toxin genes, also arranged in an alphabetical order. Only samples tested for the following 13 virulence factors were utilized [F18, F41, F4 (K88), F5 (K99), F6 (987P), AIDA, Eae, Paa, EAST1, LT, STa, STb, and Stx2e]. Samples that were not tested for at least those 13 targets were excluded from virotype analyses for consistency in presenting trends over time as well as due to the importance of those targets in *E. coli* pathogenesis. The *E. coli* PCR genotyping results of Stx1 and Stx2 were not utilized for the virotypes due to the lack of evidence on their role in *E. coli* disease causation in pigs [28]. Those 13 targets have been consistently tested since 2014, so the dataframe for virotype analyses spans from 2014 to 2024.

#### 2.3.4. Pathotype

The pathotype classification was performed based on the combination of attachment and toxin genes present in a single sample, considering the role each virulence factor plays in the *E. coli* mechanism for causing different diseases. If a virotype did not possess the combination of virulence factors that characterized a pathotype, it was described as “*Not potentially pathogenic*.” If all targets were negative, it was considered “*Negative*.” The combination of attachment and toxin genes used for each pathotype in this study is described in Table 1. Only samples tested for all the described targets in Table 1 were utilized. The pathotypes utilized in this project were ETEC, STEC, enteropathogenic *E. coli* (EPEC), and their hybrids: ETEC/STEC, ETEC/EPEC, STEC/EPEC, and ETEC/STEC/EPEC. The extraintestinal pathogenic *E. coli* (ExPEC) pathotype has also been described for pigs, causing colisepticemia, polyserositis, and urogenital infection [2]. However, ExPEC has not been included in this study as the virulence factors described for ExPEC are not part of the PCR genotyping targets used in the participating laboratories.

**Table 1:** Mapping of the virulence factors used to classify the pathotypes of *Escherichia coli*.

Pathotype	Attachment	Toxins
ETEC	*Must have at least one of:* F5 (K99), F6 (987P), F41, F4 (K88), and F18 *May have:* AIDA and Paa	*Must have at least one of:* STa, STb, and LT *May have:* EAST1 *Must not have:* STx2e
STEC	*Must have at least one of:* F5 (K99), F6 (987P), F41, F4 (K88), and F18 *May have:* AIDA and Paa	*Must have:* STx2e *May have:* EAST1 *Must not have:* STa, STb, and LT
Hybrid ETEC/STEC	*Must have at least one of:* F5 (K99), F6 (987P), F41, F4 (K88), and F18 *May have:* AIDA and Paa	*Must have:* STx2e *Must have at least one of:* STa, STb, and LT *May have:* EAST1
EPEC	*Must have:* Eae (intimin) *May have:* AIDA and Paa *Must not have:* F5 (K99), F6 (987P), F41, F4 (K88), and F18	Can be any or none.
Hybrid ETEC/EPEC	*Must have:* Eae (intimin) *Must have at least one of:* F5 (K99), F6 (987P), F41, F4 (K88), and F18 *May have:* AIDA and Paa	*Must have at least one of:* STa, STb, and LT *May have:* EAST1 *Must not have:* STx2e
Hybrid STEC/EPEC	*Must have:* F18 and Eae (intimin) *May have:* F5 (K99), F6 (987P), F41, F4 (K88), AIDA, and Paa	*Must have:* STx2e *May have:* EAST1 *Must not have:* STa, STb, and LT
Hybrid STEC/EPEC/ETEC	*Must have:* F18 and Eae (intimin) *May have:* F5 (K99), F6 (987P), F41, F4 (K88), AIDA, and Paa	*Must have:* STx2e *Must have at least one of:* STa, STb, and LT *May have:* EAST1
Not potentially pathogenic	Only attachment (not Eae) or only toxins	
Negative	None of the targets were positive	

*Note:* Pathotype definitions: Negative, no virulence factors detected; Not potentially pathogenic, virulence factor profile not associated with known pathogenic pathotypes.

Abbreviations: EPEC, enteropathogenic *Escherichia* (*E*.) *coli*; ETEC, enterotoxigenic *E. coli*; Hybrid ETEC/EPEC, hybrid enterotoxigenic and enteropathogenic *E. coli*; Hybrid ETEC/STEC, hybrid enterotoxigenic and shiga toxin‐producing *E. coli*; Hybrid STEC/EPEC, hybrid shiga toxin‐producing and enteropathogenic *E. coli*; Hybrid STEC/EPEC/ETEC, hybrid strain combining shiga toxin‐producing, enteropathogenic, and enterotoxigenic *E. coli* characteristics; STEC, shiga toxin‐producing *E. coli*.

#### 2.3.5. Potentially Pathogenic

Samples classified as pathotypes “*Not Potentially Pathogenic”* or “*Negative”* were considered “*No*” for being potentially pathogenic. All other pathotype classifications were considered “*Yes*” for being potentially pathogenic. The classification of potentially pathogenic bacteria was determined based on the specific PCR targets detected in each sample rather than on a predefined set of required targets that must be tested.

#### 2.3.6. Animal Category

A subset of samples collected between 2014 and 2025 was selected to explore *E. coli* detection trends by pig age. Samples from pigs aged 1–7 days were used to represent likely neonatal diarrhea‐associated virotypes, while those from pigs aged 20–36 days were used to represent likely post‐weaning diarrhea‐associated virotypes. Only samples tested for 13 targets (F18, F41, F4, F5, F6, AIDA, Eae, Paa, EAST1, LT, STa, STb, and Stx2e) and classified as potentially pathogenic were included in this age‐dependent analysis.

#### 2.3.7. Statistical Analysis

Changes in virulence factors, pathotypes, and potentially pathogenic trends were assessed over time. The Mann–Kendall trend test was utilized to evaluate monotonic temporal trends in yearly prevalence from 2014 to 2025 [29, 30]. The cutoff year was set at 2014 as this was the first year in which all reported virulence factors were consistently tested, including Stx1 and Stx2, which were not included in the panel before this year. The test produces the Mann–Kendall S statistic, which indicates an increasing trend (positive values), a decreasing trend (negative values), or no trend. Statistical significance was assessed using a two‐sided *p*‐value at the 95% significance level. Analyses were performed in R using the *trend* package [31].

## 3. Results

The initial dataset comprised 38,383 samples submitted for *E. coli* PCR genotyping between 2000 and 2025. After restricting the period from 2008 to 2025, 34,708 samples remained. After excluding research‐related cases, the final dataset included 30,143 samples. For the virotype analysis, 17,043 samples collected between 2014 and 2025 and tested for the 13 described targets were included.

The number of samples tested by *E. coli* PCR genotyping showed a relatively stable trend from 2008 onward, with a median of 1735 samples per year (minimum of 1140 in 2018 and maximum of 2089 in 2011). The site states that the majority of submissions came from Iowa (25%; 7537/30,143) and Minnesota (25%; 7529/30,143). As for the animal category, most samples originated from nursery pigs (35.8%; 10,780/30,143), followed by the unknown category (27.2%; 8197/30,143), suckling piglets (16.6%; 5,002/30,143), and grow‐finish pigs (15.2%; 4583/30,143). The remaining samples were from breeding herds (4.5%; 1353/30,143), replacement animals (0.5%; 146/30,143), adults (0.2%; 56/30,143), and boar studs (0.09%; 26/30,143).

### 3.1. Trends in Virulence Factors Detection

**Figure 2:**
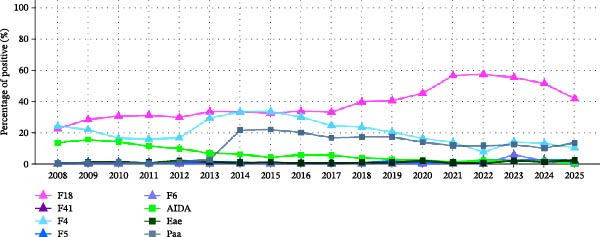
Detection of *Escherichia coli* attachment genes by PCR from 2008 to 2025.

**Figure 3:**
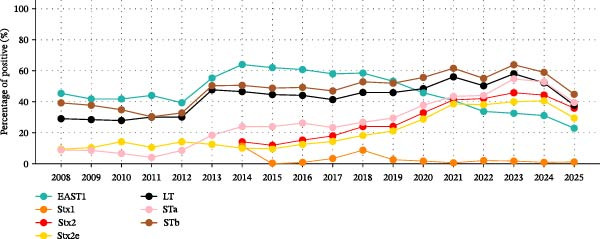
Detection of *Escherichia coli* toxin genes by PCR from 2008 to 2025.

Between 2008 and 2025, numerical changes occurred in the detection of *E. coli* virulence factors among swine samples (Figures 2 and 3). The Mann–Kendall statistics showed an increase in the detection of the virulence factors F18, F41, STa, Stx2, and Stx2e over time. The virulence factors F4, AIDA, Paa, and EAST1 showed decreased detection. No trend was observed for F5, F6, Eae, LT, STb, and Stx1 (Table S1).

**Figure 4:**
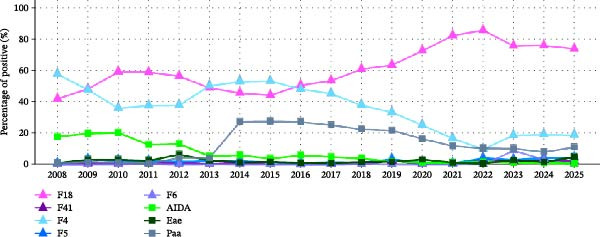
Detection of *Escherichia coli* attachment genes by PCR from 2008 to 2025 in samples classified as potentially pathogenic.

**Figure 5:**
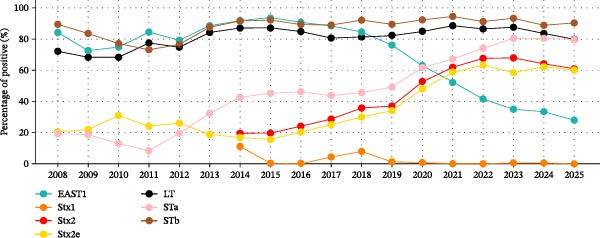
Detection of *Escherichia coli* toxin genes by PCR from 2008 to 2025 in samples classified as potentially pathogenic.

Marked shifts in the trends of virulence factors were identified within samples classified as potentially pathogenic (Figures 4 and 5). The virulence factors F18, F41, STa, Stx2, and Stx2e showed increased detection over time (*p*‐values of 0.047 or less). The virulence factors F4, AIDA, Paa, EAST1, and Stx1 showed decreased detection, and no trend was observed for F5, F6, Eae, LT, and STb (Table S2).

### 3.2. Trends in Virotypes

The virotype profile among *E. coli* samples from swine has shifted notably over the last 11 years (Table 2). The virotype F18:LT:STa:STb:Stx2e markedly increased from undetected (0%; 0/1567) in 2014 to become the most detected by 2025, representing 22.2% (257/1157). Between 2014 and 2025, notable trends shifted regarding virotypes containing F4 and F18 fimbriae. In 2014, virotypes carrying the F4 fimbriae were detected in 33.1% (519/1567) of the samples, while F18‐positive virotypes accounted for 33.8% (529/1567). By 2025, the proportion of F4‐positive virotypes decreased to 10.5% (121/1157), whereas that of F18‐positive virotypes increased to 41.9% (485/1157). These findings indicate a change in the distribution of fimbrial virulence factors over the 10‐year period, with F18 becoming more detected, while F4 detection declined.

**Table 2:** Detection of *Escherichia coli* virotypes over time.

Year	Most detected virotype	% (*n*/total)	Second most detected virotype	% (*n*/total)	Third most detected virotype	% (*n*/total)
2014	F18:EAST1:LT:STb	10.6% (166/1567)	F4:Paa:EAST1:LT:STa:STb	8.4% (132/1567)	F4:EAST1:LT:STb	8.0% (126/1567)
2015		11.3% (163/1444)		10.4% (150/1444)		6.8% (98/1444)
2016		11.7% (167/1425)		9.9% (141/1425)	F4:EAST1:LT:STa:STb	6.5% (92/1425)
2017		9.4% (117/1239)	EAST1	6.8% (84/1239)	F4:Paa:EAST1:LT:STa:STb	6.2% (77/1239)
2018		13.9% (146/1049)	F4:Paa:EAST1:LT:STa:STb	6.1% (64/1049)	F4:EAST1:LT:STb	5.4% (57/1049)
2019		11.9% (154/1298)	F18:LT:STa:STb:Stx2e	6.8% (88/1298)	F4:Paa:EAST1:LT:STa:STb	6.1% (79/1298)
2020	F18:LT:STa:STb:Stx2e	15.4% (223/1449)	F18:EAST1:LT:STb	9.5% (137/1449)	F18:EAST1:LT:STa:STb:Stx2e	6.1% (88/1449)
2021		24.1% (450/1869)		8.9% (167/1869)		6.5% (122/1869)
2022		25.1% (421/1675)		6.9% (115/1675)		6.2% (104/1675)
2023		29.9% (475/1588)		6.2% (98/1588)		6.0% (95/1588)
2024		30.2% (387/1283)		4.6% (59/1283)	EAST1	4.2% (54/1283)
2025		22.2% (257/1157)	F18:Paa:EAST1	4.8% (55/1157)		2.9% (33/1157)

*Note:* Total, total number of samples tested. *n*, number of samples that were positive.

### 3.3. Trends in Pathotypes

**Figure 6:**
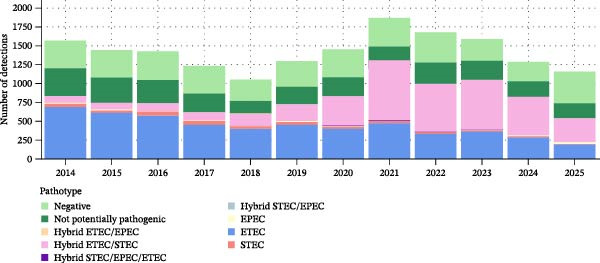
Detection of *Escherichia coli* pathotypes over the years.

Over the years 2014–2025, notable shifts were also observed in the pathotype profiles of swine *E. coli* isolates (Figure 6). The only pathotype with an increase in detection was Hybrid ETEC/STEC (*p*‐value < 0.001). The pathotypes ETEC, STEC, and Not Potentially Pathogenic showed decreased detection. No trend was observed for EPEC, Negative Hybrid ETEC/EPEC, Hybrid STEC/EPEC, and Hybrid STEC/EPEC/ETEC (Table S3).

### 3.4. Trends in Potentially Pathogenic

**Figure 7:**
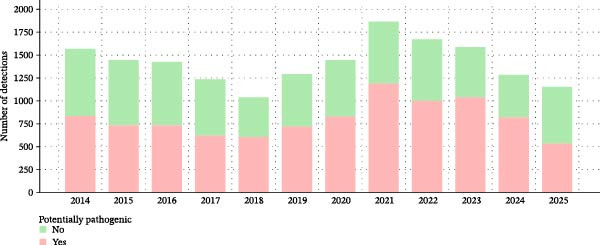
Pathogenic potential of *Escherichia coli* in swine samples over the years.

The trends of isolates classified as potentially pathogenic are represented in Figure 7. Numerically, there seems to be an increase in the detection of potentially pathogenic isolates from 2020 onward; however, it was not statistically significant (Mann–Kendall S statistic = 26; *p*‐value = 0.086).

### 3.5. Trends by Animal Category

Different virotypes have been reported in cases likely associated with neonatal diarrhea; however, the relatively small number of samples submitted for PCR genotyping (a median of 8 per year) may limit the ability to identify clear temporal trends. More recently, since 2023, the virotype F4:STa:STb has been the most frequently detected (Table S4). As for likely post‐weaning diarrhea‐associated, F18:LT:STa:STb:Stx2e, a hybrid ETEC/STEC pathotype, is the most frequently detected virotype over the past 5 years (Table 3).

**Table 3:** Detection of *Escherichia coli* virotypes in cases likely associated with post‐weaning diarrhea.

Year	Most detected virotype for likely post‐weaning diarrhea	% (*n*/total)
2014	F18:EAST1:LT:STb	18.5% (40/216)
2015	F4:Paa:EAST1:LT:STa:STb	20.8% (35/168)
2016	F18:EAST1:LT:STb	23.4% (44/188)
2017		18% (25/139)
2018		22.3% (31/139)
2019		24.7% (48/194)
2020	F18:LT:STa:STb:Stx2e	23.3% (49/210)
2021		32.5% (90/277)
2022		43.9% (125/285)
2023		42.6% (109/256)
2024		39.8% (84/211)
2025		50% (70/140)

*Note:* Total, total number of samples tested. *n*, number of samples that were positive.

## 4. Discussion

The presented study provided a comprehensive overview of *E. coli* virulence factor detection in porcine clinical isolates over the last years. This study retrieved diagnostic data from 6 major U.S. diagnostic laboratories that commonly receive porcine samples. The integration of *E. coli* PCR genotyping diagnostic data results across laboratories enabled the analysis of 30,143 samples. This significantly enhances the understanding of emerging trends and the macroepidemiology of pathogenic *E. coli* strains in the U.S. swine industry. Highlighted is the emergence of the virotype F18:LT:STa:STb:Stx2e and, consequently, the pathotype hybrid ETEC/STEC. These findings demonstrate the evolving pathogen characteristics of *E. coli* in swine samples and highlight the importance of robust monitoring systems for detecting and effectively managing diseases.

Approximately half of the samples (15,194/30,143) submitted annually were classified as potentially pathogenic in our study, underscoring the consistent epidemiological relevance of pathogenic *E. coli* strains in pig populations. Conversely, the other half (14,949/30,143) of the *E. coli* samples lacked identifiable virulence factors associated with pathogenicity, underscoring the critical role of molecular genotyping in assessing the pathogenic potential of *E. coli*. These findings highlight the importance of a comprehensive diagnostic approach, including bacteriological culture interpretation, PCR‐based virulence profiling, and histopathological evaluation, to accurately determine the involvement of *E. coli* in clinical disease and to rule out other potential pathogens [7, 32]. From 2020 onwards, an upward trend in the detection of potentially pathogenic strains emerged, surpassing the 40% detection threshold. This may indicate an increase in clinical relevance, suggesting changes in the herd health status or pathogen dynamics that merit further investigation.

The emergence of novel virotype profiles, particularly those containing F18 fimbria in combination with multiple toxin genes, is a pivotal finding of this study. Specifically, the virotype F18:LT:STa:STb:Stx2e transitioned from being undetected in 2014 to being the most prevalent by 2025. A recent study characterizing *E. coli* isolates from cases confirmed with post‐weaning diarrhea also observed the virotype F18:LT:STa:STb:Stx2e (27.71%) as the most frequently detected [15]. Its consistent detection in confirmed clinical cases lends further support to our findings. Conversely, traditionally dominant virotypes containing the F4 fimbria have experienced marked declines, reflecting shifts in epidemiological pressures that may be influenced by vaccination or genetic factors within swine populations [33–35]. This shift highlights the adaptability of pathogens and raises significant concerns for swine health management. In the age‐specific subset, virotypes carrying F18 were detected in pigs aged 20–36 days, whereas those carrying F4 were found in pigs aged 1–2 days. This distribution aligns with biological expectations as F18 receptors mature with age and typically become functional around 3 weeks of age [4]. In this study, F18 was very frequently detected in young‐age piglets, highlighting the importance of a holistic approach when investigating animal disease epidemiology. The detection of F18 in young piglets does not necessarily mean that it is causing disease. However, the pathogen may colonize the gut microbiota until receptors are developed, creating an opportunity for disease expression.

The pathotype analysis corroborated these findings, with a marked increase in hybrid ETEC/STEC samples and a corresponding decrease in ETEC ones. These shifts highlight evolving pathogen virulence profiles and were not due to an increase in submissions as the number of submissions remained consistent over the years. Even though STEC is classically associated with edema disease, a recent study demonstrated that one hybrid STEC/ETEC mainly caused severe diarrhea in post‐weaning pigs without edema disease in pigs [20]. One limitation of the *E. coli* genotyping PCR commonly used in VDLs is that it does not discriminate between F18ab and F18ac subtypes, so it is unclear if the hybrid STEC/ETEC isolates possess the F18ab subtype commonly associated with STEC or the F18ac subtype that is associated with ETEC, as this could be one factor contributing to the lack of an association with clinical edema disease. Additionally, STEC‐only strains have had a consistently low detection rate over the years, which may reflect a lower number of edema disease cases submitted to VDLs, but this needs to be elucidated further in a study that includes additional diagnostic data, including clinical history and gross and microscopic pathology.

Some limitations of this study include the lack of information on the health status of the pigs sampled and the final diagnostic confirmation. Some samples may have come from nonclinical pigs or pigs in which the cause of the disease was another pathogen. This opens an opportunity for further studies to be done applying the data structure presented in this study with a standardized diagnostic code (Dx code) nomenclature system, as previously described for PCV2/PCV3 [21]. Nevertheless, the diagnostic technique used to detect *E. coli* genotyping is based on gel‐based PCR, which is prone to frequent errors. Further developments in using real‐time PCR‐based assays could reduce the potential source of errors. An even more efficient and flexible approach would be the usage of next‐generation sequencing to screen *E. coli* isolates for the presence of genes of interest, opening an opportunity for the use of a multispecies diagnostic assay with the flexibility to be customized to detect additional genes of interest or even differentiation of genes such as F18ab and F18ac. Moreover, the standardized data structure developed for *E. coli* can serve as a foundation for future studies aiming to analyze trends in bacterial isolation and antimicrobial susceptibility patterns not only for porcine but also for other animal production species. Other applications of the data structure developed in this study include its use in future research to guide the selection of representative isolates for further characterization, such as next‐generation sequencing and the selection of virotypes for vaccine field trials.

## 5. Conclusions

This study’s comprehensive *E. coli* data integration approach enabled a broad evaluation of virulence factors detected by PCR and further classification into virotypes, predicted pathotypes, and potentially pathogenic strains. A clear transition in pathotype trends was observed, with a decline in classic ETEC strains and a substantial increase in hybrid ETEC/STEC strains, suggesting an evolving pathogenic profile in swine‐associated *E. coli*.

## Funding

This work was supported by the Swine Health Information Center, with project identification number #24‐017 and by the National Institute of Food and Agriculture, U.S. Department of Agriculture, with project identification number #2023‐67015‐39883.

## Conflicts of Interest

The authors declare no conflicts of interest.

## Supporting Information

Additional supporting information can be found online in the Supporting Information section.

## Supporting information


**Supporting Information** Table S1: Results of the Mann–Kendall test for trends in virulence factors from 2014 to 2025. Table S2: Results of the Mann–Kendall test for trends in virulence factors from potentially pathogenic isolates from 2014 to 2025. Table S3: Results of the Mann–Kendall test for trends in Pathotypes from 2014 to 2025. Table S4: Detection of Escherichia coli virotypes in cases likely associated with neonatal diarrhea.

## Data Availability

The SDRS project has a legal confidentiality agreement with participant VDLs, posing restrictions on sharing raw data publicly. The data used to generate information for this manuscript regarding *E. coli* PCR genotyping are publicly available on the SDRS webpage (https://fieldepi.org/sdrs/ and https://fieldepi.org/SDRS/EColi). Additional data may be made available upon reasonable request and approval by SDRS participant institutions. Please direct your request to the SDRS email address, sdrs@iastate.edu, and the corresponding author.
