# Supplementary material for: Emergence of Escherichia coli Hybrid Pathotype ETEC/STEC: A Macroepidemiological Approach of Molecular Characterization in the United States Swine Population
Source: Transbound Emerg Dis. 2026 Jul 1;2026:8370419. doi: 10.1155/tbed/8370419 (PMC13319902; doi:10.1155/tbed/8370419)
Supplement: Supplementary file 1 — Supporting Information Table S1: Results of the Mann–Kendall test for trends in virulence factors from 2014 to 2025. Table S2: Results of the Mann–Kendall test for trends in virulence factors from potentially pathogenic isolates from 2014 to 2025. Table S3: Results of the Mann–Kendall test for trends in Pathotypes from 2014 to 2025. Table S4: Detection of Escherichia coli virotypes in cases likely associated with neonatal diarrhea. [file TBED-2026-8370419-s001.docx]

**Supplementary Table 1.** Results of the Mann-Kendall test for trends in virulence factors from 2014 to 2025.

| **Virulence factor** | **Mann–Kendall S statistic** | **Trend** | ***p*-value** |
| --- | --- | --- | --- |
| F18 | 40 | Increasing | 0.007 |
| F41 | 30 | Increasing | 0.047 |
| STa | 48 | Increasing | 0.001 |
| Stx2 | 54 | Increasing | <0.001 |
| Stx2e | 54 | Increasing | <0.001 |
| F4 | -56 | Decreasing | <0.001 |
| AIDA | -54 | Decreasing | <0.001 |
| Paa | -48 | Decreasing | 0.001 |
| EAST1 | -64 | Decreasing | <0.001 |
| F5 | 22 | No trend | 0.150 |
| F6 | 23 | No trend | 0.128 |
| Eae | 2 | No trend | 0.945 |
| LT | 20 | No trend | 0.193 |
| STb | 24 | No trend | 0.115 |
| Stx1 | -16 | No trend | 0.304 |

**Supplementary Table 2.** Results of the Mann-Kendall test for trends in virulence factors from potentially pathogenic isolates from 2014 to 2025.

| **Virulence factor** | **Mann–Kendall S statistic** | **Trend** | ***p*-value** |
| --- | --- | --- | --- |
| F18 | 48 | Increasing | 0.001 |
| F41 | 30 | Increasing | 0.047 |
| STa | 54 | Increasing | <0.001 |
| Stx2 | 54 | Increasing | <0.001 |
| Stx2e | 54 | Increasing | <0.001 |
| F4 | -48 | Decreasing | 0.001 |
| AIDA | -54 | Decreasing | <0.001 |
| Paa | -58 | Decreasing | <0.001 |
| EAST1 | -64 | Decreasing | <0.001 |
| Stx1 | -30 | Decreasing | 0.047 |
| F5 | 20 | No trend | 0.193 |
| F6 | 18 | No trend | 0.234 |
| Eae | 18 | No trend | 0.244 |
| LT | -4 | No trend | 0.837 |
| STb | 2 | No trend | 0.945 |

**Supplementary Table 3.** Results of the Mann-Kendall test for trends in Pathotypes from 2014 to 2025.

| **Virulence factor** | **Mann–Kendall S statistic** | **Trend** | ***p*-value** |
| --- | --- | --- | --- |
| Hybrid ETEC/STEC | 56 | Increasing | <0.001 |
| Not Potentially Pathogenic | -40 | Decreasing | 0.007 |
| ETEC | -60 | Decreasing | <0.001 |
| STEC | -44 | Decreasing | 0.003 |
| Negative | -14 | No trend | 0.373 |
| EPEC | 6 | No trend | 0.732 |
| Hybrid ETEC/EPEC | 4 | No trend | 0.830 |
| Hybrid STEC/EPEC | -17 | No trend | 0.087 |
| Hybrid STEC/EPEC/ETEC | 17 | No trend | 0.239 |

**Supplementary Table 4:** Detection of *Escherichia coli* virotypes in cases likely associated with neonatal diarrhea.

| **Year** | **Most detected virotype for likely neonatal diarrhea** | **% (n/total)** |
| --- | --- | --- |
| 2014 | F4:EAST1:LT:STb | 42.9% (6/14) |
| 2015 | F4:EAST1:LT:STa:STb | 30% (3/10) |
| 2016 |  | 25% (3/12) |
| 2017 | F4:STa:STb | 57.1% (4/7) |
| 2018 | F4:EAST1:LT:STb | 37.5% (3/8) |
| 2019 | F4:Paa:EAST1:LT:STa:STb | 25% (2/8) |
| 2020 | F4:STa:STb | 50% (4/8) |
| 2021 | F4:EAST1:LT:STb | 50% (3/6) |
| 2022 | F4:STa:STb:Stx2e | 42.9% (3/7) |
| 2023 | F4:STa:STb | 14.3% (1/7) |
| 2024 |  | 62.5% (5/8) |
| 2025 |  | 50% (2/4) |

Total = total number of samples tested. n = number of samples that were positive.
